# Perturbations of the endometrial immune microenvironment in endometriosis and adenomyosis: their impact on reproduction and pregnancy

**DOI:** 10.1007/s00281-025-01040-1

**Published:** 2025-02-18

**Authors:** Jialu Shi, Qianhan Xu, Shuyi Yu, Tao Zhang

**Affiliations:** 1 Department of Obstetrics and Gynaecology, The Chinese University of Hong Kong, Hong Kong, China; 2 Joint Laboratory in Reproductive Medicine, Chinese University of Hong Kong, Sichuan University, The Chinese University of Hong Kong, Hong Kong, China

**Keywords:** Endometrial immune cells, Endometriosis, Adenomyosis, Reproduction, Pregnancy

## Abstract

The impact of endometriosis and adenomyosis on reproduction and pregnancy is significant, with both conditions linked to increased rates of infertility, poor ovarian function in women with endometriosis, and elevated pregnancy complications in those with adenomyosis. However, the underlying mechanisms remain largely unclear. Both conditions share a similar pathophysiological process characterized by the growth of ectopic endometrium, which may originate from the eutopic endometrium. Notably, surgical removal of ectopic lesions does not appear to significantly improve reproductive and pregnancy outcomes, further underscoring the importance of eutopic endometrium in these adverse effects. Emerging evidence indicates substantial differences in endometrial NK cells, macrophages, and T cells, leading to inflammatory responses in women with endometriosis and adenomyosis. These alterations may contribute not only to disease progression but also to defective endometrial receptivity, insufficient angiogenesis remodeling, impaired maternal-fetal immune tolerance, and poor placentation, thereby influencing embryo implantation and pregnancy maintenance. This provides an immunological perspective to explain the higher rates of infertility and pregnancy complications observed in affected women. Therefore, we systematically review the alterations in endometrial immune cells in women with endometriosis and adenomyosis compared to healthy controls, exploring the potential impacts of these changes on reproduction and pregnancy. This review aims to lay the groundwork for future studies on the immunopathogenesis associated with endometriosis and adenomyosis-related reproductive failure and pregnancy complications, shedding lights on the development of immunotherapeutic strategies to mitigate these adverse impacts in affected women.

## Introduction

Endometriosis and adenomyosis are two of the common gynaecological disorders, which are often considered together due to similar symptoms and pathophysiology. Both conditions are characterized by the growth of ectopic endometrial tissues: in endometriosis, this tissue is located outside the uterus, while in adenomyosis, it is found within the myometrium. The precise aetiology of both conditions is not yet fully understood, although various theories have been proposed attempting to explain them. Among these, the most widely accepted include Sampon’s theory of retrograde menstruation, which suggests that shedding endometrial tissue travel through the fallopian tubes and enters the peritoneal cavity in cases of endometriosis. In contrast, adenomyosis is thought to involve the infiltration of endometrial basal layer deep into the myometrium due to an absent or altered junctional zone.

Importantly, a high degree of association between the two conditions further suggests these two diseases may share a common origin in abnormal eutopic endometrium [1]. Indeed, several studies have shown cellular and molecular differences in the eutopic endometrium between women with endometriosis or adenomyosis and healthy controls [2, 3]. However, most studies did not clearly exclude one condition from the other or specify the comorbidity, making it uncertain how similar the changes in eutopic endometrium are between endometriosis and adenomyosis.

Emerging evidence suggests that women with endometriosis and/or adenomyosis have a higher incidence of infertility and pregnancy complications, which may be partially attributed to impaired decidualization due to changes in the eutopic endometrium [4]. In addition to the defective decidualization of stromal cells, these women exhibit significant alterations in endometrial immune cells, which not only facilitate the disease progression, as previously reviewed [5], but also negatively impact reproduction and pregnancy [6].

In healthy women, endometrial immune cells dynamically adapt to the biological events during the preparation of the endometrium for embryo implantation and subsequent decidualization, thereby supporting embryo growth thereafter. Proper immunomodulation is crucial for angiogenic remodeling, maternal immune tolerance to the semi-allogenic fetus, and successful placentation. Disruption and dysfunction of this intricate system can result in infertility, miscarriage, and other pregnancy complications [7]. However, whether the changes in endometrial immune cells in women with endometriosis increase the risk of infertility and pregnancy complications remains largely unknown. In this review, we aim to examine the endometriosis and adenomyosis-related alterations in endometrial immune cells and discuss their potential contributions to infertility and pregnancy complications.

### Increased incidence of reproductive failure and pregnancy complications in women with endometriosis and adenomyosis

Endometriosis has been reported to affect approximately 5–10% of reproductive-aged women worldwide and increase the risk of female infertility twofold [8]. The pathophysiology of endometriosis impacts reproduction at nearly every stage, including gametes, fertilization, embryo implantation, and placentation. These effects are further supported by clinical data. Women affected by endometriosis, particularly endometrioma, often exhibit significantly lower ovarian reserve function and fecundity [9]. Similarly, multiple meta-analyses consistently show that women with endometriosis undergoing in vitro fertilization-embryo transfer (IVF-ET) tend to have significantly lower number of oocytes retrieved, as well as decreased fertilization and implantation rates (Table 1). This suggests endometriosis adversely affects both the quantity and quality pf oocytes. In contrast to the effects on oocytes, the influence of endometriosis on pregnancy rate, miscarriage rate and live birth rate is not consistent across these meta-analyses (Table 1). Similarly, the effects of endometriosis on pregnancy complications, such as preterm birth, small for gestational age, preeclampsia, cesarean delivery, stillbirth and neonatal death, show variability. However, the increased risk of placenta previa in women with endometriosis is relatively well recognized. A meta-analysis conducted by Gasparri et al. suggested that endometriosis alone may act as an independent risk factor for placenta previa [10]. Nevertheless, these meta-analyses did not conduct subgroup analysis based on specific types of endometriosis [11, 12], such as deep infiltrating endometriosis, which may have a more severe impact on reproductive and pregnancy outcomes.

**Table 1:** Summarized meta-analysis of pregnancy complications in women with endometriosis

Reference	Publication year	Study dates	No. of studies	No. of cases	Mode of conception	No. of oocytes retrieved	Fertilization rate	Implantation rate	Pregnancy rate	Miscarriage rate	Live birth rate	Placenta previa	Other obstetric complications
Barnhart et al. [140]	2002	1980–1999	22	6760	IVF	↓^a^, ↓^d^	↓^a^, ↓^d^	↓^a^, ↔^d^	↓^a^, ↓^d^	-	-	-	-
Harb et al. [11]	2013	~ 2012	27	8984	IVF	-	↓^b^, ↔^c^	↓^c^, ↔^b^	↓^c^, ↔^b^	-	↔^c^, ↔^b^	-	-
Hamdan et al. [12]	2015	1980–2014	36	529,454	IVF/ICSI	↓^a^, ↓^c^	-	-	↓^a^, ↓^c^	↔^a^	↔^a^, ↓^c^	-	-
Hamdan et al. [20]	2015	1980–2014	33	-	IVF/ICSI	↓^a^	-	-	↔^a^	↔^a^	↔^a^	-	-
Horton et al. [141]	2019	1980–2018	63	-	ART/ SC	↓^a^,	↓^a^, ↓^b^	↓^b^	-	↑^a^	-	-	Preterm birth ↑^a^, cesarean section ↑^a^, neonatal unit admission following delivery ↑^a^
Wang et al. [142]	2021	~ 2019	28	2,409,064	ART/ SC	-	-	-	-	↑^a^	-	↑^a^	Preterm birth ↑^a^, gestational hypertension ↑^a^, cesarean section ↑^a^, preeclampsia ↑^a^, placental abruption ↔^a^
Huang et al. [143]	2020	~ 2020	-	-	ART/ SC	-	-	-	-	↑^a (SC)^ , ↔^a(ART)^	-	↑^a^	Preterm birth ↑^a^, stillbirth ↑^a^, antepartum hemorrhage ↑^a^, postpartum hemorrhage ↑^a^, placental abruption ↔^a^, pre-eclampsia ↔^a^, gestational diabetes ↔^a^, low birthweight ↔^a^, intrauterine growth restriction ↔^a^
Bruun et al. [144]	2018	1950–2017	17	-	-	-	-	-	-	-	-	-	Preterm birth ↑^a^, small for gestational age ↑^a^
Lalani et al. [145]	2018	1990–2017	33	3,280,488	ART/ SC	-	-	-	-	-	-	↑^a (ART, SC)^	Preterm birth ↑^a (ART, SC)^, cesarean section ↑^a (SC)^, low birth weight ↑^a (SC)^, pre-eclampsia ↑^a (mixed)^, gestational diabetes ↑^a (mixed)^, gestational cholestasis ↑^a (mixed)^, antepartum hemorrhage ↑^a (mixed)^
Gasparri et al. [10]	2018	~ 2018	5	8007	ART	-	-	-	-	-	-	↑^a^	Placental abruption ↔^a^
Qu et al. [146]	2022	~ 2021	70	-	IVF/ICSI	↓^a^	-	↓^a^	-	-	↔^a^, ↔^c^	↑^a^	Postpartum hemorrhage ↑^a^, preterm birth ↔^a^, preeclampsia ↔^a^ postpartum hemorrhage ↑^a^, small for gestational age ↔^a^
Nagase et al. [147]	2022	~ 2021	28	4,719,258	-	-	-	-	-	-	-	-	Instrumental delivery ↑^a^, cesarean section ↑^a^, postpartum hemorrhage ↔^a^

No., number; ↓, risk significantly decreased; ↑, risk significantly increased; ↔, no difference in risk; -, not applicable; ^a^, endometriosis vs. control; ^b^, mild endometriosis (stage I-II) vs. control; ^c^, severe endometriosis (stage III-IV) vs. control; ^d^, severe endometriosis (stage III-IV) vs. mild endometriosis (stage I-II); IVF, in vitro fertilization; ICSI, intracytoplasmic sperm injection; ART, assisted reproduction technology; SC, spontaneous conception

The estimated prevalence of adenomyosis is hard to obtain and probably underestimated, ranging from 8.8 to 61.5% of hysterectomy patients [13]. Women with adenomyosis also experience a higher incidence of infertility (about 24.4% of infertile women over 40 years old and 22% in infertile women less than 40) [14]. Multiple meta-analyses (Table 2) indicate that women with adenomyosis undergoing IVF-ET retrieve a similar number of oocytes compared to those without the condition. However, affected women exhibit significantly lower pregnancy and live birth rates, along with notably higher incidences of miscarriage, preterm birth, preeclampsia, fetal malpresentation, cesarean section, and postpartum hemorrhage [15–18]. These adverse effects are also observed in women with spontaneous conception [19]. It is clear that adenomyosis poses a substantial obstacle to achieving a healthy pregnancy and warrants increased attention of obstetric care providers.

**Table 2:** Summarized meta-analysis of pregnancy complications in women with adenomyosis

Reference	Publication year	Study dates	No. of papers	No. of cases	Mode of conception	No. of oocytes retrieved	Implantation rate	Pregnancy rate	Miscarriage rate	Live birth rate	Preterm birth	Preeclampsia	Small for gestational age	Other obstetric complications
Vercellini et al. [15]	2014	1999–2012	9	1865	IVF	-	-	↓	↑	↓	-	-	-	-
Younes et al. [16]	2017	~ 2012	11	2054	IVF	-	↓	↓	↑	↓	-	-	-	-
Bruun et al. [144]	2018	1950–2017	4	-	-	-	-	-	-	-	↑	-	↑	-
Razavi et al. [17]	2019	2006–2018	6	9742	-	-	-	-	-	-	↑	↑	↑	Fetal malpresentation ↔
Horton et al. [141]	2019	1980–2018	11	-	ART/ SC	-	-	↓	↑	↓	↑	↑	↑	Cesarean section ↑
Huang et al. [143]	2020	~ 2020	-	-	ART	-	-	-	↑	-	-	-	-	-
Nirgianakis et al. [18]	2021	~ 2020	17	-	ART	-	-	↓	↑	-	↑	↑	↑	Cesarean section↑, fetal malpresentation ↑, postpartum hemorrhage ↑
Cozzolino et al. [148]	2022	~ 2020	22	-	IVF	↔	-	↓	↑	↓	-	-	-	-

No., number; ↓, risk significantly decreased; ↑, risk significantly increased; ↔, no difference in risk; -, not applicable; IVF, in vitro fertilization; ART, assisted reproduction technology; SC, spontaneous conception

Unfortunately, surgical and hormonal treatments do not seem to significantly reverse these adverse effects of endometriosis and adenomyosis on reproduction and pregnancy [20, 21]. Therefore, it is crucial to uncover the mechanisms underlying these negative effects to promote the development of novel therapeutic strategies.

### Endometrial macrophages

Macrophages, derived from monocytes, are essential components of the immune system to adapt and respond to various physiological and pathological stimuli. The term “macrophage” comes from the ancient Greek words “makros” (large) and “phagein” (to eat), reflecting their role in engulfing and digesting cellular debris, pathogens, and foreign substances. Macrophages can be broadly classified into two main types based on their ontogeny and function: M1 and M2. M1 macrophages, also known as classically activated macrophages, are pro-inflammatory, and involved in pathogen elimination and tissue damage. They secrete a range of pro-inflammatory cytokines contributing to inflammation and pathogen clearance. In contrast, M2 macrophages, or alternatively activated macrophages, are associated with tissue repair, immune regulation, and the resolution of inflammation. Various markers are used to identify the functions and phenotypes of macrophages, as summarized in Table 3. These cells are distributed throughout the body, where they maintain tissue homeostasis, regulate inflammation, and orchestrate immune responses. These versatile cells are highly adaptable and can adjust their functions based on the local microenvironment. In the female reproductive tract, they are pivotal for ensuring the proper functioning of the reproductive system, including menstrual cycle changes, embryo implantation, pregnancy maintenance, and the initiation of parturition. Changes in macrophages associated with conditions such as endometriosis and adenomyosis may impact reproduction and pregnancy.

**Table 3:** The markers of human and mouse macrophages

Marker	Species	Structure	Function	Expression Profile	M1/M2/Pan
CD68	Human, Mouse	Transmembrane glycoprotein	Phagocytosis, lysosomal marker	Cell surface, intracellular	Pan
CD80	Human, Mouse	Transmembrane protein	Co-stimulatory molecule for T-cell activation	Cell surface	M1
CD86	Human, Mouse	Transmembrane protein	Co-stimulatory molecule for T-cell activation	Cell surface	M1
iNOS	Human, Mouse	Enzyme	Produces nitric oxide in response to inflammation	Intracellular	M1
TNF-α	Human, Mouse	Cytokine	Pro-inflammatory cytokine	Soluble	M1
CD206	Human, Mouse	Transmembrane protein	Mannose receptor, endocytosis	Cell surface	M2
Arg1	Human, Mouse	Enzyme	Involved in polyamine synthesis, M2 marker	Intracellular	M2
CD163	Human, Mouse	Transmembrane protein	Scavenger receptor, anti-inflammatory	Cell surface	M2
IL-10	Human, Mouse	Cytokine	Anti-inflammatory cytokine	Soluble	M2
CD11b	Human, Mouse	Integrin	Phagocytosis, cell adhesion, migration	Cell surface	Pan
F4/80	Mouse	Transmembrane glycoprotein	Macrophage-specific marker	Cell surface	Pan
MHC II	Human, Mouse	Transmembrane protein	Antigen presentation	Cell surface	M1
CD14	Human, Mouse	Co-receptor	LPS recognition, part of the TLR4 complex	Cell surface	Pan

### Dynamics and plastic functions of endometrial/decidual macrophages during menstrual cycle and pregnancy

**Fig. 1:**
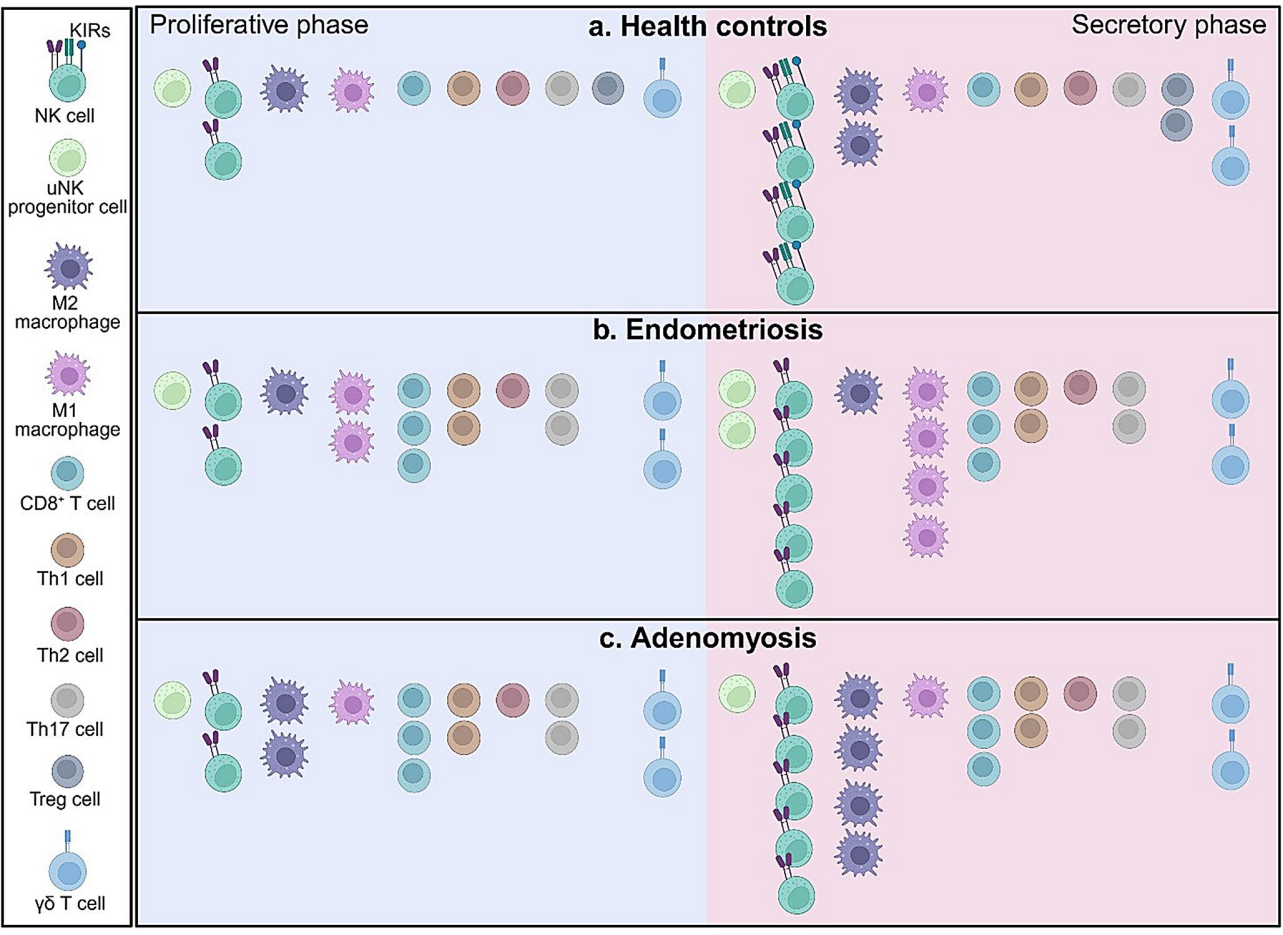
Dynamics of endometrial immune cell counts during menstrual cycle in endometriosis and adenomyosis. Compared to healthy controls, women with endometriosis exhibit a significant increase in NK cells, uNK progenitor cells, M1 macrophages, CD8^+^ T cells, Th1 cells, Th17 cells and γδT cells in the endometrium. Similarly, women with adenomyosis also showed a significant increase in NK cells, M2 macrophages, CD8^+^ T cells, Th1 cells, Th17 cells and γδT cells in the endometrium. KIRs: killer-immunoglobulin-like receptors. Created with Biorender. com

During the menstrual cycle, macrophages exhibit distinct fluctuations of population and activity within the endometrium. In the proliferative phase, CD68^+^ or CD163^+^ macrophages constitute 1–2% of endometrial cells, increasing to 1–5% in the early-to-mid secretory phase, then rising dramatically to 7% in the late secretory phase, and peaking at 6–15% during the pre-menstrual phase [22, 23]. The majority of endometrial macrophages are of the M2 phenotype [24]. These accumulated macrophages are typically found as single cells or in clusters within the glandular lumens of the superficial endometrium [25], where they play crucial roles in tissue repair and angiogenesis. Furthermore, the remarkable increase in macrophages during the secretory phase supports the endometrium’s transitions into the decidua by promoting stromal cell differentiation into decidual cells (decidualization) through the secretion of factors such as IL-10 and TGF-β. M2 macrophages also play a role in regulating extracellular matrix remodeling, which is essential for successful decidualization and placentation. In contrast, the frequency of CD68^+^IL-10^−^iNOS^+^ M1 macrophages in the secretory endometrium (lower than 20%) is significantly lower compared to the proliferative phase (about 30%) [26]. This reduction in M1 macrophages during the secretory phase is crucial for fostering endometrial receptivity by creating a favorable immune environment essential for successful implantation (Fig. 1).

**Fig. 2:**
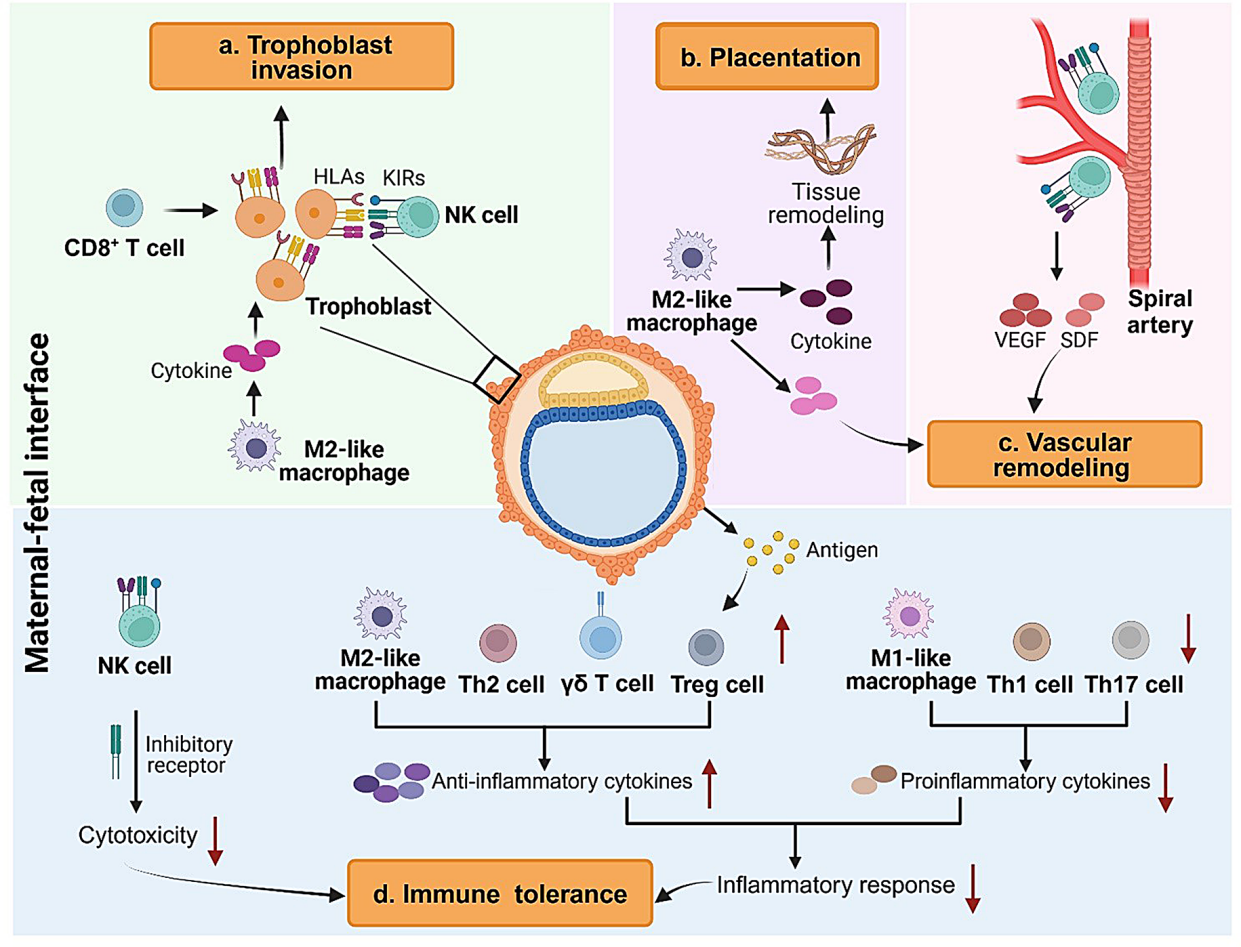
Roles of endometrial immune cells in establishing and maintaining early pregnancy. At the maternal-fetal interface, endometrial immune cells contribute to pregnancy in four main ways: (**a**) NK cells, M2-like macrophages and CD8^+^ T cells regulate trophoblast invasion during early pregnancy through KIRs and cytokine secretion, respectively; (**b**) M2-like macrophages facilitate placental development by regulating tissue remodeling via cytokine secretion; (**c**) NK cells and M2-like macrophages contribute to vascular remodeling through producing cytokines; (**d**) NK cell cytotoxicity is reduced, and there is simultaneous down-regulation of the inflammatory response by macrophages and T cells, thereby promoting immune tolerance. HLA: human leukocyte antigen; KIRs: killer-immunoglobulin-like receptors; VEGF: vascular endothelial growth factor; SDF: stromal cell-derived factor. Created with Biorender. com

Decidual macrophages exhibit a spectrum of activation states shaped by the dynamic microenvironment. They are classified into decidual M1-like and M2-like subtypes based on their functional tendencies rather than strict polarization. Decidual M1-like macrophages are primarily involved in immune defense against potential infections, while decidual M2-like macrophages dominate from early pregnancy through the third trimester, playing crucial roles in tissue remodeling, immune tolerance, and promoting trophoblast invasion. During parturition, recognized as an inflammatory event, M1-like macrophages become the predominant subset, secreting pro-inflammatory cytokines and chemokines that promote uterine contractions and the breakdown of decidual tissues, facilitating the expulsion of the fetus and placenta (Fig. 2).

This cyclical and adaptive behavior of macrophages underscores their versatile and indispensable roles in female reproduction, from regulating endometrial receptivity and supporting fetal development to orchestrating the complex process of pregnancy.

### Unique changes of endometrial macrophages in endometriosis and their potential contributions to infertility and miscarriage

Endometriosis is associated with significant alterations in the number and function of macrophages in eutopic endometrium [27]. Research has demonstrated an increased density of macrophages, particularly during the proliferative phase, in women with endometriosis compared to controls [28]. Additionally, the functional phenotypes undergo significant shifts. In women with endometriosis, the M2 subpopulation is reduced across all phases compared with controls [28]. The reduced presence of CD163^+^ macrophages, with an area under the curve (AUC) of 0.833, suggests its potential as a diagnostic marker for endometriosis [28]. Consistently, RNA sequencing analysis of fluorescence-activated cell-sorted macrophages has revealed an increased presence of pro-inflammatory M1 macrophages in the endometrium of women with endometriosis, compared to healthy controls [29]. Utilizing mass cytometry, researchers have identified an increased abundance of CD91^+^ macrophages in the eutopic endometrium of individuals with endometriosis. These macrophages play a crucial role in the efferocytosis of apoptotic cells during endometrial shedding through the calreticulin/CD91 pathway. Although their numbers are elevated in endometriosis, these macrophages exhibit defective phagocytic capacity, potentially due to the altered inflammatory microenvironment, which contributes to the survival of endometrial cells during shedding [30].

Furthermore, changes in the endometrial macrophages correlate with the stage of endometriosis. M1 macrophages are more prevalent in early-stage endometriosis (stages I–II), while M2 macrophages increase in advanced-stage disease (stages III–IV) [31]. Paradoxically, despite their typical anti-inflammatory nature, M2 macrophages in the endometriotic endometrium exhibit a pro-inflammatory phenotype, likely driven by the altered microenvironment in endometriosis [30]. The persistent inflammatory milieu of endometriosis seems to override their typical immunosuppressive nature, driving them to adopt a paradoxical pro-inflammatory behavior [30]. Pathways enriched in advanced stages, such as TGF-β, PI3K/AKT/mTOR, IFN-γ signaling, along with metabolic reprogramming, may further support this polarization [31]. However, the underlying mechanism and remain unclear.

Altered macrophages in both eutopic and ectopic endometrium may impact infertility in several mechanisms. The increase in M1 macrophages in the eutopic endometrium may contribute to defective endometrial receptivity for embryo implantation as the dominance of M2 macrophages plays an essential role in this process through cytokine regulation and immune cell interactions. However, research specifically addressing the issue is limited. In contrast, more evidence suggests that activated macrophage and their cytokine secretions, including TNF-α, GM-CSF, IL-1, and IL-6, in the peritoneal fluid are pivotal in negatively affecting oocyte quality and impairing embryo development [32, 33]. These inflammatory cytokines may impact the environment in the fallopian tube and endometrial cavity, leading to oxidative stress that disrupts fertilization and early embryo development, though the precise mechanisms remain unclear. Additionally, macrophages from the peritoneal fluid of infertile patients with endometriosis had higher sperm phagocytosis compared to those from infertile women without endometriosis, suggesting a direct detrimental effect on fertilization [34]. This heightened inflammatory status within the pelvic cavity likely plays a central role in reducing fertility [35].

**Fig. 3:**
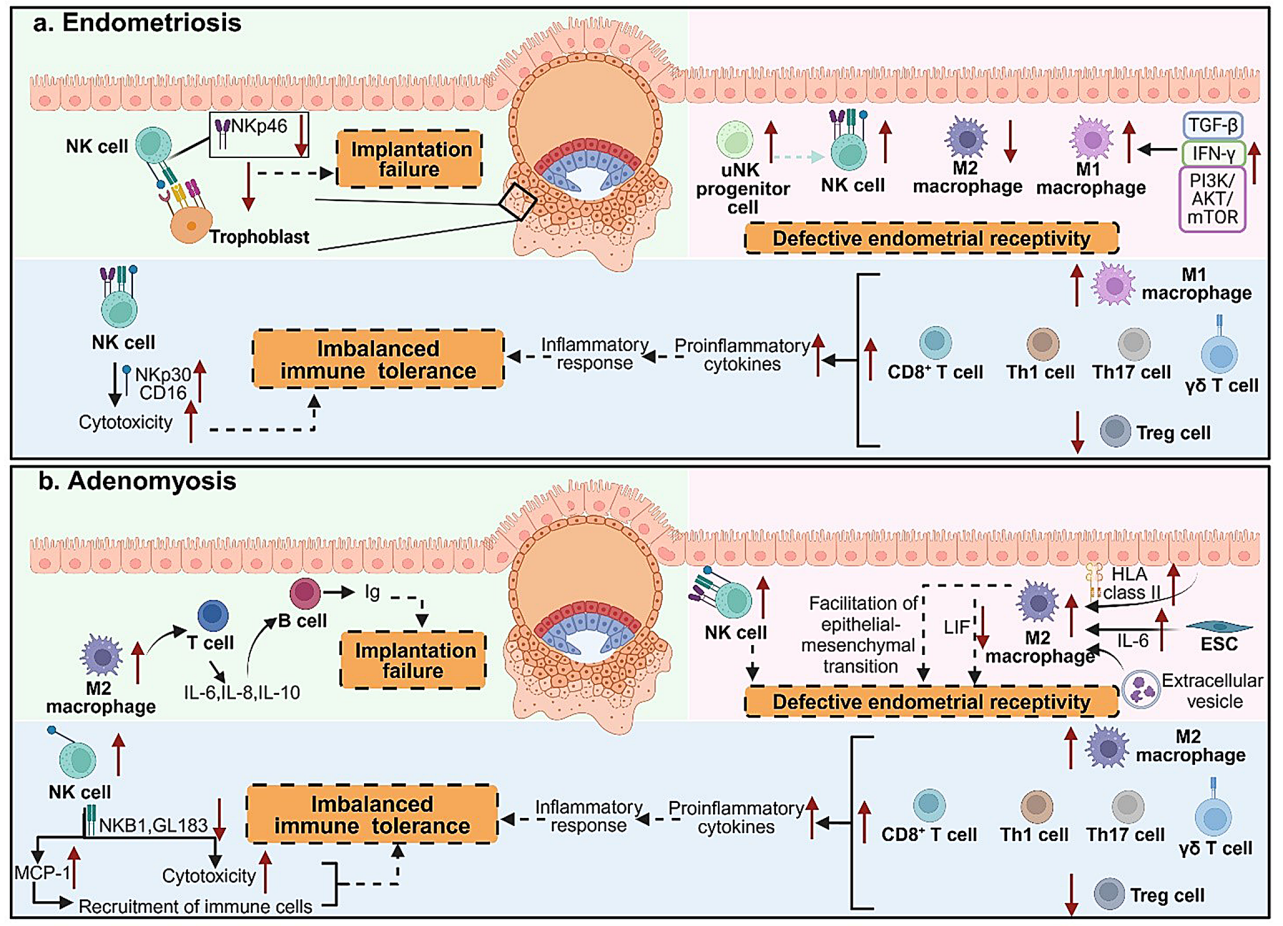
Possible mechanisms by which alterations in endometrial immune cells in endometriosis/adenomyosis contribute to reproductive failure. (**a**) In endometriosis, embryo implantation may fail due to the downregulation of NK cell activation receptors, resulting in diminished interaction with trophoblast cells. Concurrently, an abnormal increase in the number of uNK progenitor cells, NK cells, and M1 macrophages may lead to defective endometrial receptivity. Additionally, enhanced NK cell cytotoxicity and increased secretion of pro-inflammatory cytokines by M1 macrophages and T cells combine to lead to an excessive inflammatory response. (**b**) In adenomyosis, there is an abnormal increase in M2 macrophages, which may lead to implantation failure by regulating the activation of T cells and B cells. Furthermore, the elevated number of M2 macrophages and NK cells can impair endometrial receptivity. Moreover, increased NK cell cytotoxicity, their recruitment of immune cells, and increased secretion of proinflammatory cytokines by M2 macrophages and T cells collectively drive a pro-inflammatory response that disrupts maternal immune tolerance. HLA: human leukocyte antigen; ESC: endometrial stromal cell; LIF: leukemia inhibitory factor. Created with Biorender. com

In addition to infertility, excessive pro-inflammatory M1 macrophages, combined with a weakened anti-inflammatory M2 profile may increase the risk of miscarriage in women with endometriosis by impairing immune tolerance [36]. This imbalance in macrophages disrupts the modulation of the local immune environment, leading to an overactive inflammatory response that may interfere with crucial processes such as trophoblast invasion and placental development. However, decidual macrophages, different from endometrial macrophages, have distinct roles in supporting early pregnancy [37]. The specific changes in decidual macrophages in women with endometriosis have yet to be fully revealed (Fig. 3a).

### Distinctive changes of endometrial macrophages in adenomyosis and their potential contributions to infertility

Several studies have confirmed that macrophages are highly enriched in the eutopic and ectopic endometrium of patients with adenomyosis, aggregating within the superficial endometrial glands [38, 39]. Recently, a single-cell RNA sequencing study revealed that immune cells made up 24% of the total cell population in the eutopic endometrium of adenomyosis patients during the follicular phase, with macrophages and monocytes comprising 4% of the total cell population, identified by CD74 and human leukocyte antigen (HLA)-DRA expression [40]. Compared to women without adenomyosis, the density of CD163^+^ M2 macrophages in the eutopic endometrium of patients with severe diffuse or local adenomyosis is significantly increased [38, 41]. In contrast, immune cells represent only 13% of the cell population in the ectopic endometrial tissue, where macrophages and monocytes are the most abundant, contributing to 6% [40]. Genes associated with immune response and macrophage activation, such as CD74, HLA-DRB1, HLA-DRA, S100A6, and NFKBIA, are significantly overexpressed in the adenomyotic epithelium [40, 42]. These findings indicate that macrophages are activated and may lead to inflammation in adenomyosis, but this issue remains underexplored.

The increased presence of M2 macrophages in adenomyosis appears to play a pivotal role in driving the condition’s associated infertility. While M2 macrophages are typically involved in tissue repair and anti-inflammatory processes, their excessive polarization in adenomyosis leads to pathological tissue fibrosis and chronic inflammation, which undermines normal endometrial function. This dysregulated activity in adenomyosis is primarily driven by signals from the eutopic endometrial cells. For instance, the study conducted by Yang et al. [43] demonstrated that endometrial stromal cells (ESCs) from adenomyotic tissue exhibited elevated IL-6 mRNA expression, leading to M2 polarization. Similarly, Hu et al. illustrated how adenomyotic tissue directly influences macrophage behavior through extracellular vesicles, which also induce M2 polarization [44]. The polarized macrophages could, in turn, facilitate epithelial-mesenchymal transition, a biological process where epithelial cells lose their cell-cell adhesion properties and gain migratory and invasive characteristics typical of mesenchymal cells [44]. This transition enables the epithelial cells to break through the basement membrane, promoting the spread of ectopic endometrial tissue into the myometrium, and enhancing the invasiveness of adenomyotic lesions [39, 44]. Moreover, M2 macrophages activate signaling pathways such as TGF-β1/Smad3 and IL-6/JAK2/STAT3, promoting cell growth and proliferation within adenomyotic lesions, further contributing to the progression and severity of the condition [45].

The overactivity of M2 macrophages disrupts the key signaling molecules necessary for embryo implantation, such as leukemia inhibitory factor (LIF), which is crucial for creating an implantation-receptive endometrium [46]. In a healthy endometrium, the proper immune environment supports optimal LIF production, which regulates surface glycan structures on epithelial cells. However, in adenomyosis, the overactivation of M2 macrophages, combined with the ongoing inflammatory response, may impair LIF production or its regulatory functions, indirectly compromising the endometrial receptivity needed for successful implantation [46, 47]. Research has suggested that macrophage-derived-LIF significantly decreased in both the endometrium and uterine flushing fluid during the window of implantation in women with adenomyosis-related infertility [46, 47].

Additionally, endometrial macrophages may influence embryo implantation through interactions with other immune cells, such as T cells and B cells, further exacerbating the inflammatory milieu. In adenomyotic endometrium, high expression of HLA class II can activate macrophages, which in turn stimulate T cells to secrete IL-6, IL-8, and IL-10 [5, 48]. These cytokines then stimulate B cells to produce immunoglobulins (Ig), creating an immune response that potentially hinder embryo implantation [5]. These findings suggest that abnormal cytokine secretion and immune cell interactions involving endometrial macrophages may create an immunological ‘vicious circle’ that exacerbates adenomyosis-related infertility [49]. However, the precise mechanisms by which endometrial macrophages contribute to infertility in adenomyosis remain poorly understood and warrant further investigation (Fig. 3b).

### Uterine NK cells

Natural killer (NK) cells are defined as effector innate lymphoid cells produced by progenitor cells in the bone marrow, with the capacity to recognize and eliminate distressed cells [50]. Human NK cells are typically classified based on the expression of two surface molecules: CD56 and CD16. Consequently, human peripheral NK (pNK) cells can be divided into two main subpopulations: CD56^dim^CD16^+^ pNK cells, which comprise approximately 90% of the population, and CD56^bright^CD16^−^ pNK cells, which account for about 10% [51]. Unlike the majority of pNK cells, uterine NK (uNK) cells generally express CD56 at high levels while lacking CD16. Furthermore, uNK cells express adhesion molecules that facilitate tissue residency, which phenotypically differ from those of CD56^bright^CD16^−^ pNK cells. Recent studies using single-cell RNA sequencing demonstrate that the heterogeneity of uNK cells is much more complex than a simple classification based on CD56 and CD16 expression [52, 53]. However, further studies are needed to validate these new categories.

As the predominant immune cells during the implantation window and early pregnancy [54], uNK cells play a crucial role in embryo implantation and placentation. Disruptions in their density or function have been linked to immune etiology of reproductive failure [55]. Emerging studies have reported significant alterations in uNK cells in women with endometriosis and adenomyosis, which may increase the risk of reproductive failure.

### Dynamic changes and plastic functions of uNK during reproduction

The morphology of uNK cells changes with menstrual cyclicity. In the proliferative phase, uNK cells are characterized by small granules; while in the secretory phase after ovulation, they gradually enlarge and possess larger granules [56]. Throughout the menstrual cycle, the density of uNK cells among total leukocytes reaches 30–40% in the proliferative phase, increases further to 60% in the secretory phase, peaks at 70% of the total leukocytes in early gestation, and then declines from mid-pregnancy [51, 57, 58]. This variation highlights their importance in embryo implantation and early pregnancy (Fig. 1).

NK cell receptors (NKRs) regulate NK cell functions, primarily through killer-immunoglobulin-like receptors (KIRs), which are transmembrane receptors featuring both activating and inhibitory isoforms that can interact with fetal HLAs. It is crucial to maintain a functional balance between activating and inhibitory KIRs for embryo implantation and placentation in early pregnancy [59]. Most uNK and decidual NK (dNK) cells share a unique repertoire of NKR, with a notable preference for KIR2D expression [60]. However, emerging evidence indicates that dNK cells differ from uNK cells by expressing significantly lower frequencies of KIRs, especially KIR2DS1, KIR2DL2L3S2 and KIR2DL2S2, while exhibiting considerably higher frequencies of activation receptors NKG2D, NKp30, NKp46 and CD244 [61]. This reduction in KIR expression may be attributed to increased interactions between these receptors and their respective HLA ligands on trophoblasts [62]. Conversely, the upregulation of receptors on dNK cells could be a result of elevated IL-15 levels derived from stromal cells [63]. The activation of these upregulated receptors can lead to elevated production of chemokines, cytokines and angiogenic factors, such as vascular endothelial growth factor (VEGF) and stromal cell-derived factor (SDF), thereby facilitating vascular growth and trophoblast invasion [64] (Fig. 2).

Taken together, the plasticity of NK cells leads to the heterogeneity of various subtypes, as well as the diversity of functions, which lays an important foundation for successful pregnancy.

### Changes of uNK cells in endometriosis and their potential contributions to reproductive failure

Similar to healthy women, those with endometriosis exhibit a significant increase in uNK cells during the secretory phase [65]. The dramatic increase of uNK cells might from three sources: (1) in situ proliferation of mature uNK cells in uterus [66]; (2) migration of peripheral NK cells [67]; and (3) maturation and differentiation of NK progenitor cells [68]. However, patients with severe endometriosis show significantly higher numbers of CD56^+^ uNK cells in the mid-luteal phase compared to healthy women [69]. Additionally, the number of uNK progenitor cells (i.e. uNK cells at developmental stages) is noticeably increased in women with endometriosis [68], indicating a dysfunction in the in situ development of mature uNK cells and potential dysregulation of their functions [68, 70]. Accumulated evidence demonstrates that women with repeated implantation failure (RIF), typically defined as failure to implant after three consecutive transfers of high-quality embryos, and those with recurrent miscarriage (RM), usually defined as two or three consecutive miscarriage before 20 or 24 weeks of gestation, both show an increased percentage of uNK cells. However, further studies are needed to determine whether the increase in uNK cells contributes to the high incidence of infertility and miscarriage in women with endometriosis. The expression of NKp46 and the NKp46^+^/CD56^+^ cell ratio is both found significantly decreased in patients with severe endometriosis compared with healthy women [69]. Meanwhile, low expression of NKp46 in uNK cells has also been demonstrated in women with reproductive disorders such as RIF and RM [71, 72]. Given the involvement of NKp46 in uNK cell activation/maturation and angiogenic functions during pregnancy in mice [73], its inadequacy may imply a deficiency in the number and function of uNK cells in patients with severe endometriosis, potentially leading to their inadequate pregnancy support. There are also data suggesting that the expression of NKp30 (a natural cytotoxicity receptor of uNK cells) and its ligand BAG6 is upregulated in uNK cells of the eutopic endometrium of endometriosis [65]. It may lead to the increased cytotoxicity of these uNK cells, which is detrimental to reproduction (Fig. 3a).

### Changes of uNK cells in adenomyosis and their potential contributions to reproductive failure

At present, there was a paucity of publication describing the relationship between uNK cells and adenomyosis. No significant difference in the number of CD56^+^ uNK cells has been reported in the eutopic endometrium of patients with adenomyosis compared with controls in two previous studies. However, a subgroup analysis of the adenomyosis cohort revealed that the number of uNK cells did not increase in women with mild adenomyosis while it was elevated in the eutopic endometrium of women with severe adenomyosis (either diffuse or adenomyoma type) during the late luteal phase (cycle days 22–26) [5, 38, 74, 75]. As stated before, the increased concentration of uNK cells in adenomyosis may also affect endometrial receptivity thereby contributing to infertility and miscarriage, which warrants further studies to substantiate.

Yang et al. recruited 10 women with adenomyosis (5 in the luteal phase) and 12 women without adenomyosis (6 in the luteal phase) to compare the expression of KIRs. They found reduced expression of KIRs, including NKB1 and GL183, on uNK cells in the eutopic endometrium from patients with adenomyosis [75], which may be a compensated response to increased cytotoxicity of uNK cells, potentially resulting in lower fertility in these patients.

Additionally, a prospective case-control study found that the expression of monocyte chemoattractant protein-1 (MCP-1), a cytokine produced by uNK cells that triggers the migration of immune cells to target tissues, was significantly increased in endometrial tissues of patients with adenomyosis compared with controls during the implantation window after ovarian stimulation [76]. The aggregation of immune cells may lead to excessive proinflammatory response at the maternal-fetal interface, potentially resulting in low clinical pregnancy rates and high miscarriage rates in patients with adenomyosis [5] (Fig. 3b).

Overall, the current understanding of the impact of uNK cell alterations on reproductive failure in endometriosis and adenomyosis remains limited, warranting further investigation.

### Endometrial T cells

Throughout menstrual cycle and pregnancy, fluctuations in female hormones orchestrate changes in T cell numbers and functions to meet various physiological needs, balancing immune tolerance–essential for a successful pregnancy–with immune activation needed for protection against infection [77]. However, this balance can be disrupted in endometriosis and adenomyosis, potentially leading to reproductive disorders and pregnancy complications.

### Dynamics and plastic functions of endometrial T cells during reproduction

A variety of uterine T cell subsets regulate immune responses during the menstrual cycle, embryo implantation and pregnancy. These endometrial T cells primarily include CD4^+^ helper T cells, CD8^+^ T cells, regulatory T cells (Treg), and γδ T cells. The composition of T cells in the endometrium shows a similar proportion of CD4^+^ T cells and CD8^+^ T cells, unlike peripheral blood, where CD4^+^ T cells comprise two-thirds of the T cell population [78]. Decidual T cells exhibit a more differentiated state, with a lower proportion of CD45RA^+^naive T cells compared to peripheral and endometrial T cells [79]. The number of endometrial T cells do not significantly fluctuate during the menstrual phase [80], but the increasing numbers of NK cells and macrophages contribute to a reduced proportion of T lymphocyte [58].

The majority of endometrial CD8^+^ T cells are CD45RO^+^memory T cells [78]. During the proliferative phase, CD8^+^ T cells exhibit high cytolytic activity to recognize and eliminate abnormal or infected cells [81]. In the luteal phase, CD8^+^ T cells lose their cytolytic function to avoid attacking the allogeneic embryo [81]. In early pregnancy, decidual CD8^+^T cells are capable of promoting the invasive capacity of trophoblast cells [82].

Unlike CD8^+^ T cells, CD4^+^ T helper cells modulate the immune response and contribute to endometrial homeostasis by secreting cytokines. Based on their cytokine profiles, endometrial CD4^+^ T helper cells can be divided into Th1, Th2 and Th17 subsets. Th1 cells produce pro-inflammatory cytokines, Th2 cells secrete anti-inflammatory cytokines, and Th17 cells uniquely produce the pro-inflammatory cytokine IL-17 [83, 84]. Additionally, Tregs, characterized by the transcription factor Foxp3 and surface markers CD25, play a crucial role in maintaining immune tolerance by suppressing excessive immune responses [84]. The dynamic balance between local Th1 and Th2 cells, as well as Th17 and Tregs reflects the inflammatory response, which is crucial for maintaining immune tolerance at the maternal-fetal interface [83].

During the luteal phase, endometrial Tregs significantly increase compared to the proliferative phase, indicating a shift to an anti-inflammatory state that is crucial for inducing immune tolerance and preventing inflammatory damage to the embryo [85]. After embryo implantation, Tregs respond to fetal antigens, proliferate significantly, and become enriched in the decidua [86]. During late pregnancy, Treg clonal expansion increases compared to early stage, suggesting a strong requirement for antigen-specific tolerance during late gestation [86]. In contrast, Th17 cells remain stable throughout pregnancy [87].

Another important T cell subset is γδ T cells, identified by the γδ T-cell receptor and divided into two main sub-populations: Vδ1 and Vδ2 [88]. Compared to the proliferative phase, γδT cell are also significantly higher in the luteal phase and during pregnancy [89]. The Vδ1 cells are known to secrete Th2-type cytokines, thereby creating a specialized immune microenvironment that supports embryo implantation and early pregnancy (Fig. 2).

### Endometrial T cell alterations in endometriosis

Various endometrial T cells exhibit significant changes in women with endometriosis compared to healthy or non-endometriosis controls. Firstly, endometrial CD3^+^ T cells significantly increase during the proliferative phase, although they are similar in the luteal phase in patients with endometriosis [90]. Secondly, the proportion of CD8^+^ T_EM_ cells, primarily to recognize and eliminates infected cells, is significantly higher in the eutopic endometrium across all menstrual phases in endometriosis [91, 92]. Additionally, increased secretion of IL-17a from Th17 cells leads to the accumulation of neutrophils and proliferation of endometriotic cell, resulting in persistent inflammation [93]. Consistently, single-cell transcriptomic analysis of endometrium from women with endometriosis revealed elevated levels of proinflammatory cytokines [94]. Furthermore, one study demonstrates a reduction of Foxp3^+^ cells in the peri-implantation endometrium of patients with endometriosis [95], a finding supported by recent studies [96] (Fig. 3a).

### Endometrial T cell alterations in adenomyosis

Research on the local immune microenvironment in adenomyosis is relatively limited compared to endometriosis, but the pattern of pro-inflammatory response is similar. Increased numbers of CD3^+^ T cells, CD4 ^+^ and CD8^+^ T cells and γδT cells have been reported in the eutopic endometrium of patients with adenomyosis compared to controls [90, 97, 98]. Additionally, decreased Foxp3 and increased IL-17 A expression levels are observed in the eutopic endometrium of women with adenomyosis, and these changes are positively associated with the severity of dysmenorrhea [99] (Fig. 3b).

### Potential contributions of T cell alterations to reproductive failure in endometriosis and adenomyosis

Accumulated evidence suggests that disruptions in endometrial T cell dynamics, both before and after pregnancy, are correlated with reproductive failure. Excessive CD8^+^ T cell activity in the mid-luteal phase endometrium and during pregnancy have also been found in women with unexplained RM [100]. Additionally, an imbalance in endometrial Th cell populations, particularly a shift towards Th1 or Th17 dominance and insufficient Tregs has been linked to unexplained RIF and RM [85, 101, 102]. Consistently, decreased Treg cells have been observed in the decidual of spontaneous abortion compared to induced abortion [103]. Furthermore, decreased Tregs are predictive markers for early pregnancy failure in infertile patients [104]. Similarly, women with endometriosis and adenomyosis exhibit elevated CD8^+^T cells [92, 97], γδT cells [98], Th1 and Th17 cytokine profile in the mid-luteal phase endometrium [93, 99], alongside decreased Tregs [96, 103]. It is plausible to hypothesize that these changes may contribute to reproductive failure. However, direct evidence of this causal effect is lacking, as many studies have not conducted subgroup analysis based on the history of reproductive failure or reproductive outcomes. Additionally, no animal studies have been conducted to demonstrate the causal effects. Further investigation is warranted to understand their contributions to reproductive failure in endometriosis and adenomyosis (Fig. 3).

### Other immune cells

In addition to NK cells, macrophages, and T cells, other endometrial immune cells, including dendritic cells (DC), B cells, and neutrophils also play a role in shaping the immune microenvironment for embryo implantation and placentation. However, these cells are rarely studied in women with endometriosis and adenomyosis. Limited evidence suggests that DC maturation is defective in women with endometriosis, with a higher density of immature DC [105]. While the changes in endometrial B cells in women with endometriosis are controversial, serum antibody titers against the endometrium are significantly higher in these women [106]. Additionally, the number of neutrophils and their attractant IL-8 have been reported to be substantially increased in women with endometriosis [29, 107]. In contrast, neutrophils are debated in limited reports of adenomyosis, with most studies indicating decreased levels of IL-8 in the eutopic endometrium of women with adenomyosis compared to controls [5]. Overall, it remains unclear whether these changes affect reproduction and pregnancy.

### Potential upstream mediators driving immune cell alterations in endometriosis and adenomyosis

The underlying causes of endometrial immune cell alterations that contribute to the inflammatory response in endometriosis and adenomyosis remain unclear due to a lack of direct evidence. We speculate that factors driving chronic inflammation in the microenvironment may act as upstream mediators.

### Chronic endometritis

Chronic endometritis (CE) is a persistent inflammatory condition of the endometrium, characterized by the presence of plasma cells. It is usually asymptomatic and been reported to be related with infertility and miscarriage due to chronic inflammation [108]. Interestingly, the prevalence of CE was significantly higher in patients with endometriosis and adenomyosis compared with those without these conditions [109–112]. Furthermore, in infertile patients with endometriosis, CE is linked to decreased cumulative pregnancy rate and live birth rate [113], and increased risk of pregnancy complications [114]. These findings reveal a strong association between CE and endometriosis/adenomyosis.

The local chronic inflammation environment in women with CE induces significant alterations in the composition of endometrial immune cells. These changes are characterized by an increase in CD68^+^macrophages [115], elevated CD56^+^ uNK cells during the mid-luteal phase [116, 117], and higher levels of CD3^+^T cells, CD8^+^ T cells, Th1 cells, and Foxp3^+^ Treg cells [116]. In contrast, Th2 cells are decreased [115, 118]. Hence, it is plausible that CE in the context of endometriosis or adenomyosis contributes to the disruption of endometrial immune cells.

The most common cause of CE is infection triggered by pathogenic microorganisms, often occurring alongside dysbiosis. The *Lactobacillus* predominates in a healthy uterus and inhibits the colonization and infection of pathogens, while non-*Lactobacillus-*dominant microbiotas have been associated with adverse reproductive outcomes [119]. Compared with healthy women, patients with endometriosis exhibit reduced *Lactobacillus* abundance in the uterus, along with increased microbiota diversity [120, 121]. Similarly, women with adenomyosis are less likely to show a *Lactobacillus-*dominant endometrial microbiota [122]. The pathogenic microbiome can initiate immune response mediated by antigen presenting cells. Additionally, microbiota metabolites may regulate immune cell function [123]. Therefore, the changed endometrial microbiota in CE might be another driving factor affecting endometrial immune cells, which requires further studies.

### Elevated local estrogen

Both endometriosis and adenomyosis are characterized by elevated estrogen levels in the endometriotic lesions [124–126]. Estrogen receptors are not expressed on most endometrial leukocytes [127], with the exception of γδT cells [128]. Studies have shown that estrogen can directly enhance IL-17 expression in endometrial γδT cells [128]. Although research on the impact of estrogen on endometrial immune cells is limited, some studies have demonstrated that estrogen can regulate the development and function of lymphocytes either through direct interaction or indirectly via products from stromal and epithelial cells that express hormone receptors [129, 130]. Several in vivo studies have highlighted estrogen’s effects on immune cells, including promoting the proliferation of Foxp3^+^Treg cells [131], and enhancing the activity of IFN-producing Th1 cells through estrogen receptor α on hematopoietic cells [132]. Therefore, the elevated estrogen concentration in adenomyosis and endometriosis may contribute to immune cell alterations, although direct evidence supporting this hypothesis remains lacking.

### Epigenetic changes and inflammation

Epigenetics is the study of how genetic information is transmitted to offspring without changes to DNA sequence, through processes like DNA methylation, histone modifications, chromatin remodeling, RNA transcriptional changes [133]. Abnormal gene expression in immune cells resulting from epigenetic alterations can lead to a loss of immune tolerance, inflammation, and autoimmunity [134]. Current evidence strongly suggests that epigenetic mechanisms play a significant role in the pathogenesis of endometriosis, including hypermethylation at the ends of chromosomes in endometriotic stromal cells, increased activity of histone deacetylases (HDACs) in endometriotic cells, altered miRNA expression patterns, and nuclear receptor modulation [135]. Similarly, the etiology of adenomyosis is linked to epigenetic dysregulation, with notable changes such as elevated expression of HDAC1 and HDAC3 in both eutopic and ectopic endometria, as well as promoter hypermethylation of the progesterone receptor B isoform (PR-B) [136]. Abnormal expression of inflammation-related factors due to epigenetic alterations has been reported in both endometriosis and adenomyosis. For example, excessive production of prostaglandin E_2_ and cytokines such as TNFα, IL-1β, IL-6, IL-8, IFNγ, and MCP-1 is observed in endometriosis lesions [137], while low expression of PR-B (acting as an anti-inflammatory agent) is found in both conditions [138, 139]. These epigenetic changes and the resulting inflammation in the endometrium suggest that epigenetic mechanisms may contribute to the inflammatory environment characteristic of endometriosis and adenomyosis. Although direct studies linking epigenetic alterations to endometrial immune cell dysfunction in these diseases are limited, this hypothesis provides a promising direction for further research.

## Conclusions

Despite strong evidence indicating an increased incidence of infertility in women with endometriosis, as well as higher rates of infertility, miscarriage, and pregnancy complications in women with adenomyosis, research on the underlying mechanisms has not kept pace. Immunological adaptation is critical for the success of each stage during reproduction and pregnancy. Women with endometriosis and adenomyosis exhibit significant alterations in endometrial immune cells, compared to healthy controls. Furthermore, these changes are not always consistent between endometriosis and adenomyosis, which may explain clinically different impacts they have on reproduction and pregnancy. Nevertheless, the alterations in endometrial immune cells are somewhat comparable to those in women with RIF and RM. Thus, it is plausible to hypothesize that these changes may contribute to a higher risk of reproductive disorders and pregnancy complications. To advance this field, further studies are needed to characterize endometriosis and adenomyosis-specific endometrial immune cell profiles and to identify immune targets for the development of targeted therapies in the future.

## Data Availability

There is no additional data associated with this manuscript.

## References

[1] Kunz G, Beil D, Huppert P, Noe M, Kissler S, Leyendecker G (2005) Adenomyosis in endometriosis–prevalence and impact on fertility. Evidence from magnetic resonance imaging. Hum Reprod 20:2309–2316 15919780 10.1093/humrep/dei021

[2] Xiang Y, Sun Y, Yang B, Yang Y, Zhang Y, Yu T, Huang H, Zhang J, Xu H (2019) Transcriptome sequencing of adenomyosis eutopic endometrium: a new insight into its pathophysiology. J Cell Mol Med 23:8381–8391 31576674 10.1111/jcmm.14718 PMC6850960

[3] Lessey BA, Kim JJ (2017) Endometrial receptivity in the eutopic endometrium of women with endometriosis: it is affected, and let me show you why. Fertil Steril 108:19–27 28602477 10.1016/j.fertnstert.2017.05.031 PMC5629018

[4] Pirtea P, de Ziegler D, Ayoubi JM (2023) Endometrial receptivity in adenomyosis and/or endometriosis. Fertil Steril 119:741–745 36914148 10.1016/j.fertnstert.2023.03.004

[5] Bourdon M, Santulli P, Jeljeli M, Vannuccini S, Marcellin L, Doridot L, Petraglia F, Batteux F, Chapron C (2021) Immunological changes associated with adenomyosis: a systematic review. Hum Reprod Update 27:108–129 33099635 10.1093/humupd/dmaa038

[6] Freitag N, Baston-Buest DM, Kruessel JS, Markert UR, Fehm TN, Bielfeld AP (2022) Eutopic endometrial immune profile of infertility-patients with and without endometriosis. J Reprod Immunol 150:103489 35149274 10.1016/j.jri.2022.103489

[7] Zhang T, Shen HH, Qin XY, Li MQ (2022) The metabolic characteristic of decidual immune cells and their unique properties in pregnancy loss. Immunol Rev 308:168–186 35582842 10.1111/imr.13085

[8] Zondervan KT, Becker CM, Missmer SA (2020) Endometriosis. N Engl J Med 382:1244–1256 32212520 10.1056/NEJMra1810764

[9] Sanchez AM, Vanni VS, Bartiromo L, Papaleo E, Zilberberg E, Candiani M, Orvieto R, Viganò P (2017) Is the oocyte quality affected by endometriosis? A review of the literature. J Ovarian Res 10:43 28701212 10.1186/s13048-017-0341-4 PMC5508680

[10] Gasparri ML, Nirgianakis K, Taghavi K, Papadia A, Mueller MD (2018) Placenta previa and placental abruption after assisted reproductive technology in patients with endometriosis: a systematic review and meta-analysis. Arch Gynecol Obstet 298:27–34 29602980 10.1007/s00404-018-4765-x

[11] Harb HM, Gallos ID, Chu J, Harb M, Coomarasamy A (2013) The effect of endometriosis on in vitro fertilisation outcome: a systematic review and meta-analysis. BJOG 120:1308–1320 23834505 10.1111/1471-0528.12366

[12] Hamdan M, Omar SZ, Dunselman G, Cheong Y (2015) Influence of endometriosis on assisted reproductive technology outcomes: a systematic review and meta-analysis. Obstet Gynecol 125:79–88 25560108 10.1097/AOG.0000000000000592

[13] Upson K, Missmer SA (2020) Epidemiology of adenomyosis. Semin Reprod Med 38:89–107 33105509 10.1055/s-0040-1718920 PMC7927213

[14] Puente JM, Fabris A, Patel J, Patel A, Cerrillo M, Requena A, Garcia-Velasco JA (2016) Adenomyosis in infertile women: prevalence and the role of 3D ultrasound as a marker of severity of the disease. Reprod Biol Endocrinol 14:60 27645154 10.1186/s12958-016-0185-6 PMC5029059

[15] Vercellini P, Consonni D, Dridi D, Bracco B, Frattaruolo MP, Somigliana E (2014) Uterine adenomyosis and in vitro fertilization outcome: a systematic review and meta-analysis. Hum Reprod 29:964–977 24622619 10.1093/humrep/deu041

[16] Younes G, Tulandi T (2017) Effects of adenomyosis on in vitro fertilization treatment outcomes: a meta-analysis. Fertil Steril 108: 483– 90.e3 10.1016/j.fertnstert.2017.06.025 28865548

[17] Razavi M, Maleki-Hajiagha A, Sepidarkish M, Rouholamin S, Almasi-Hashiani A, Rezaeinejad M (2019) Systematic review and meta-analysis of adverse pregnancy outcomes after uterine adenomyosis. Int J Gynaecol Obstet 145:149–157 30828808 10.1002/ijgo.12799

[18] Nirgianakis K, Kalaitzopoulos DR, Schwartz ASK, Spaanderman M, Kramer BW, Mueller MD, Mueller M (2021) Fertility, pregnancy and neonatal outcomes of patients with adenomyosis: a systematic review and meta-analysis. Reprod Biomed Online 42:185–206 33191131 10.1016/j.rbmo.2020.09.023

[19] Hashimoto A, Iriyama T, Sayama S, Nakayama T, Komatsu A, Miyauchi A, Nishii O, Nagamatsu T, Osuga Y, Fujii T (2018) Adenomyosis and adverse perinatal outcomes: increased risk of second trimester miscarriage, preeclampsia, and placental malposition. J Matern Fetal Neonatal Med 31:364–369 28110584 10.1080/14767058.2017.1285895

[20] Hamdan M, Dunselman G, Li TC, Cheong Y (2015) The impact of endometrioma on IVF/ICSI outcomes: a systematic review and meta-analysis. Hum Reprod Update 21:809–825 26168799 10.1093/humupd/dmv035

[21] Taylor HS, Kotlyar AM, Flores VA (2021) Endometriosis is a chronic systemic disease: clinical challenges and novel innovations. Lancet 397:839–852 33640070 10.1016/S0140-6736(21)00389-5

[22] Jabbour HN, Kelly RW, Fraser HM, Critchley HO (2006) Endocrine regulation of menstruation. Endocr Rev 27:17–46 16160098 10.1210/er.2004-0021

[23] Salamonsen LA, Zhang J, Brasted M (2002) Leukocyte networks and human endometrial remodelling. J Reprod Immunol 57:95–108 12385836 10.1016/s0165-0378(02)00011-6

[24] Jensen AL, Collins J, Shipman EP, Wira CR, Guyre PM, Pioli PA (2012) A subset of human uterine endometrial macrophages is alternatively activated. Am J Reprod Immunol 68:374–386 22882270 10.1111/j.1600-0897.2012.01181.x PMC3468696

[25] Russell P, Sacks G, Tremellen K, Gee A (2013) The distribution of immune cells and macrophages in the endometrium of women with recurrent reproductive failure. III: further observations and reference ranges. Pathology 45:393–401 23619588 10.1097/PAT.0b013e328361429b

[26] Tsao FY, Wu MY, Chang YL, Wu CT, Ho HN (2018) M1 macrophages decrease in the deciduae from normal pregnancies but not from spontaneous abortions or unexplained recurrent spontaneous abortions. J Formos Med Assoc 117:204–211 28465068 10.1016/j.jfma.2017.03.011

[27] Zhang T, De Carolis C, Man GCW, Wang CC (2018) The link between immunity, autoimmunity and endometriosis: a literature update. Autoimmun Rev 17:945–955 30107265 10.1016/j.autrev.2018.03.017

[28] Berbic M, Schulke L, Markham R, Tokushige N, Russell P, Fraser IS (2009) Macrophage expression in endometrium of women with and without endometriosis. Hum Reprod 24:325–332 19049988 10.1093/humrep/den393

[29] Vallvé-Juanico J, Houshdaran S, Giudice LC (2019) The endometrial immune environment of women with endometriosis. Hum Reprod Update 25:564–591 31424502 10.1093/humupd/dmz018 PMC6737540

[30] Vallve-Juanico J, Santamaria X, Vo KC, Houshdaran S, Giudice LC (2019) Macrophages display proinflammatory phenotypes in the eutopic endometrium of women with endometriosis with relevance to an infectious etiology of the disease. Fertil Steril 112:1118–1128 31843088 10.1016/j.fertnstert.2019.08.060 PMC6944306

[31] Poli-Neto OB, Meola J, Rosa ESJC, Tiezzi D (2020) Transcriptome meta-analysis reveals differences of immune profile between eutopic endometrium from stage I-II and III-IV endometriosis independently of hormonal milieu. Sci Rep 10:313 31941945 10.1038/s41598-019-57207-y PMC6962450

[32] Kolanska K, Alijotas-Reig J, Cohen J, Cheloufi M, Selleret L, d’Argent E, Kayem G, Valverde EE, Fain O, Bornes M, Darai E, Mekinian A (2021) Endometriosis with infertility: a comprehensive review on the role of immune deregulation and immunomodulation therapy. Am J Reprod Immunol 85:e13384 33278837 10.1111/aji.13384

[33] Miller JE, Ahn SH, Monsanto SP, Khalaj K, Koti M, Tayade C (2017) Implications of immune dysfunction on endometriosis associated infertility. Oncotarget 8:7138–7147 27740937 10.18632/oncotarget.12577 PMC5351695

[34] Martinez-Roman S, Balasch J, Creus M, Fabregues F, Carmona F, Vilella R, Vanrell JA (1997) Immunological factors in endometriosis-associated reproductive failure: studies in fertile and infertile women with and without endometriosis. Hum Reprod 12:1794–1799 9308814 10.1093/humrep/12.8.1794

[35] Halis G, Arici A (2004) Endometriosis and inflammation in infertility. Ann N Y Acad Sci 1034:300–315 15731321 10.1196/annals.1335.032

[36] Ma H, Cai S, Yang L, Wang L, Ding J, Li L, Li H, Huang C, Diao L (2022) How do pre-pregnancy endometrial macrophages contribute to pregnancy? J Reprod Immunol 154:103736 36113384 10.1016/j.jri.2022.103736

[37] Sheng YR, Hu WT, Shen HH, Wei CY, Liu YK, Ma XQ, Li MQ, Zhu XY (2022) An imbalance of the IL-33/ST2-AXL-efferocytosis axis induces pregnancy loss through metabolic reprogramming of decidual macrophages. Cell Mol Life Sci 79:173 35244789 10.1007/s00018-022-04197-2 PMC11073329

[38] Tremellen KP, Russell P (2012) The distribution of immune cells and macrophages in the endometrium of women with recurrent reproductive failure. II: adenomyosis and macrophages. J Reprod Immunol 93:58–63 22209314 10.1016/j.jri.2011.12.001

[39] An M, Li D, Yuan M, Li Q, Zhang L, Wang G (2017) Different macrophages equally induce EMT in endometria of adenomyosis and normal. Reproduction 154:79–92 28495851 10.1530/REP-17-0174

[40] Yildiz S, Kinali M, Wei JJ, Milad M, Yin P, Adli M, Bulun SE (2023) Adenomyosis: single-cell transcriptomic analysis reveals a paracrine mesenchymal-epithelial interaction involving the WNT/SFRP pathway. Fertil Steril 119:869–882 36736810 10.1016/j.fertnstert.2023.01.041 PMC11257082

[41] Stratopoulou CA, Cussac S, d’Argent M, Donnez J, Dolmans MM (2023) M2 macrophages enhance endometrial cell invasiveness by promoting collective cell migration in uterine adenomyosis. Reprod Biomed Online 46:729–738 36792417 10.1016/j.rbmo.2023.01.001

[42] Li Q, Shi J, Yi D, Li X, Gu Z, Yan H, Leng J (2024) The pathogenesis of endometriosis and adenomyosis: insights from single-cell RNA sequencingdagger. Biol Reprod 110:854–865 38386960 10.1093/biolre/ioae032

[43] Yang JH, Wu MY, Chang DY, Chang CH, Yang YS, Ho HN (2006) Increased interleukin-6 messenger RNA expression in macrophage-cocultured endometrial stromal cells in adenomyosis. Am J Reprod Immunol 55:181–187 16451352 10.1111/j.1600-0897.2005.00363.x

[44] Hu Y, Yuan M, Cheng L, Wang G (2023) Extracellular vesicles contribute to EMT in adenomyosis by inducing macrophage polarizationdagger. Biol Reprod 108:584–596 36721984 10.1093/biolre/ioad015

[45] An M, Li D, Yuan M, Li Q, Zhang L, Wang G (2017) Interaction of macrophages and endometrial cells induces epithelial-mesenchymal transition-like processes in adenomyosis. Biol Reprod 96:46–57 28395325 10.1095/biolreprod.116.144071

[46] Nakamura H, Jasper MJ, Hull ML, Aplin JD, Robertson SA (2012) Macrophages regulate expression of alpha1,2-fucosyltransferase genes in human endometrial epithelial cells. Mol Hum Reprod 18:204–215 22053055 10.1093/molehr/gar070

[47] Xiao Y, Sun X, Yang X, Zhang J, Xue Q, Cai B, Zhou Y (2010) Leukemia inhibitory factor is dysregulated in the endometrium and uterine flushing fluid of patients with adenomyosis during implantation window. Fertil Steril 94:85–89 19361790 10.1016/j.fertnstert.2009.03.012

[48] Baka S, Frangou-Plemenou M, Panagiotopoulou E, Makrakis E, Kaltsakas G, Hassiakos D, Kondi-Pafiti A (2011) The expression of human leukocyte antigens class I and II in women with endometriosis or adenomyosis. Gynecol Endocrinol 27:419–424 20569098 10.3109/09513590.2010.495429

[49] Ota H, Igarashi S, Hatazawa J, Tanaka T (1998) Is adenomyosis an immune disease? Hum Reprod Update 4:360–367 9825851 10.1093/humupd/4.4.360

[50] Vivier E, Rebuffet L, Narni-Mancinelli E, Cornen S, Igarashi RY, Fantin VR (2024) Natural killer cell therapies. Nature 626:727–736 38383621 10.1038/s41586-023-06945-1

[51] Díaz-Hernández I, Alecsandru D, García-Velasco JA, Domínguez F (2021) Uterine natural killer cells: from foe to friend in reproduction. Hum Reprod Update 27:720–746 33528013 10.1093/humupd/dmaa062

[52] Lai ZZ, Wang Y, Zhou WJ, Liang Z, Shi JW, Yang HL, Xie F, Chen WD, Zhu R, Zhang C, Mei J, Zhao JY, Ye JF, Zhang T, Li MQ (2022) Single-cell transcriptome profiling of the human endometrium of patients with recurrent implantation failure. Theranostics 12:6527–6547 36185612 10.7150/thno.74053 PMC9516226

[53] Vento-Tormo R, Efremova M, Botting RA, Turco MY, Vento-Tormo M, Meyer KB, Park JE, Stephenson E, Polański K, Goncalves A, Gardner L, Holmqvist S, Henriksson J, Zou A, Sharkey AM, Millar B, Innes B, Wood L, Wilbrey-Clark A, Payne RP, Ivarsson MA, Lisgo S, Filby A, Rowitch DH, Bulmer JN, Wright GJ, Stubbington MJT, Haniffa M, Moffett A, Teichmann SA (2018) Single-cell reconstruction of the early maternal-fetal interface in humans. Nature 563:347–353 30429548 10.1038/s41586-018-0698-6 PMC7612850

[54] King A, Burrows T, Verma S, Hiby S, Loke YW (1998) Human uterine lymphocytes. Hum Reprod Update 4:480–485 10027599 10.1093/humupd/4.5.480

[55] Von Woon E, Greer O, Shah N, Nikolaou D, Johnson M, Male V (2022) Number and function of uterine natural killer cells in recurrent miscarriage and implantation failure: a systematic review and meta-analysis. Hum Reprod Update 28:548–582 35265977 10.1093/humupd/dmac006 PMC9247428

[56] Spornitz UM (1992) The functional morphology of the human endometrium and decidua. Adv Anat Embryol Cell Biol 124:1–99 1561944 10.1007/978-3-642-58099-4

[57] Male V, Moffett A (2023) Natural killer cells in the human uterine mucosa. Annu Rev Immunol 41:127–151 36630598 10.1146/annurev-immunol-102119-075119

[58] Flynn L, Byrne B, Carton J, Kelehan P, O’Herlihy C, O’Farrelly C (2000) Menstrual cycle dependent fluctuations in NK and T-lymphocyte subsets from non-pregnant human endometrium. Am J Reprod Immunol 43:209–217 10836250 10.1111/j.8755-8920.2000.430405.x

[59] Wasilewska A, Grabowska M, Moskalik-Kierat D, Brzoza M, Laudański P, Garley M (2023) Immunological aspects of infertility-the role of KIR receptors and HLA-C antigen. Cells 13:59 10.3390/cells13010059 PMC10778566 38201263

[60] Feyaerts D, Kuret T, van Cranenbroek B, van der Zeeuw-Hingrez S, van der Heijden OWH, van der Meer A, Joosten I, van der Molen RG (2018) Endometrial natural killer (NK) cells reveal a tissue-specific receptor repertoire. Hum Reprod 33:441–451 29447367 10.1093/humrep/dey001

[61] Feyaerts D, Benner M, Comitini G, Shadmanfar W, van der Heijden OWH, Joosten I, van der Molen RG (2024) NK cell receptor profiling of endometrial and decidual NK cells reveals pregnancy-induced adaptations. Front Immunol 15:1353556 38571943 10.3389/fimmu.2024.1353556 PMC10987737

[62] Hackmon R, Pinnaduwage L, Zhang J, Lye SJ, Geraghty DE, Dunk CE (2017) Definitive class I human leukocyte antigen expression in gestational placentation: HLA-F, HLA-E, HLA-C, and HLA-G in extravillous trophoblast invasion on placentation, pregnancy, and parturition. Am J Reprod Immunol 77 10.1111/aji.12643 28185362

[63] Sanchez-Correa B, Bergua JM, Pera A, Campos C, Arcos MJ, Bañas H, Duran E, Solana R, Tarazona R (2017) In Vitro Culture with Interleukin-15 leads to expression of activating receptors and recovery of natural killer cell function in Acute myeloid leukemia patients. Front Immunol 8:931 28824651 10.3389/fimmu.2017.00931 PMC5545593

[64] Vacca P, Cantoni C, Prato C, Fulcheri E, Moretta A, Moretta L, Mingari MC (2008) Regulatory role of NKp44, NKp46, DNAM-1 and NKG2D receptors in the interaction between NK cells and trophoblast cells. Evidence for divergent functional profiles of decidual versus peripheral NK cells. Int Immunol 20:1395–1405 18815119 10.1093/intimm/dxn105

[65] Drury JA, Parkin KL, Coyne L, Giuliani E, Fazleabas AT, Hapangama DK (2018) The dynamic changes in the number of uterine natural killer cells are specific to the eutopic but not to the ectopic endometrium in women and in a baboon model of endometriosis. Reprod Biol Endocrinol 16:67 30021652 10.1186/s12958-018-0385-3 PMC6052567

[66] Kane N, Kelly R, Saunders PT, Critchley HO (2009) Proliferation of uterine natural killer cells is induced by human chorionic gonadotropin and mediated via the mannose receptor. Endocrinology 150:2882–2888 19196802 10.1210/en.2008-1309 PMC2709965

[67] Carlino C, Stabile H, Morrone S, Bulla R, Soriani A, Agostinis C, Bossi F, Mocci C, Sarazani F, Tedesco F, Santoni A, Gismondi A (2008) Recruitment of circulating NK cells through decidual tissues: a possible mechanism controlling NK cell accumulation in the uterus during early pregnancy. Blood 111:3108–3115 18187664 10.1182/blood-2007-08-105965

[68] Thiruchelvam U, Wingfield M, O’Farrelly C (2016) Increased uNK progenitor cells in women with endometriosis and infertility are Associated with low levels of endometrial stem cell factor. Am J Reprod Immunol 75:493–502 26791471 10.1111/aji.12486

[69] Alimoradi Fard M, Ghafourian M, Mousavi-Salehi A, Moramazi F, Ranjbari N (2024) Immunohistochemical evaluation of NKP46 receptor expression and the number of NK Cells in the Endometrium of patients with endometriosis. Iran J Immunol 21:27–36 38375785 10.22034/iji.2024.100630.2715

[70] Granick JL, Simon SI, Borjesson DL (2012) Hematopoietic stem and progenitor cells as effectors in innate immunity. Bone Marrow Res 2012:165107 22762001 10.1155/2012/165107 PMC3385697

[71] Takeyama R, Fukui A, Mai C, Yamamoto M, Saeki S, Yamaya A, Shibahara H (2021) Co-expression of NKp46 with activating or inhibitory receptors on, and cytokine production by, uterine endometrial NK cells in recurrent pregnancy loss. J Reprod Immunol 145:103324 33930666 10.1016/j.jri.2021.103324

[72] Fukui A, Funamizu A, Yokota M, Yamada K, Nakamua R, Fukuhara R, Kimura H, Mizunuma H (2011) Uterine and circulating natural killer cells and their roles in women with recurrent pregnancy loss, implantation failure and preeclampsia. J Reprod Immunol 90:105–110 21632120 10.1016/j.jri.2011.04.006

[73] Felker AM, Chen Z, Foster WG, Croy BA (2013) Receptors for non-MHC ligands contribute to uterine natural killer cell activation during pregnancy in mice. Placenta 34:757–764 23806179 10.1016/j.placenta.2013.06.004 PMC3742434

[74] Jones RK, Bulmer JN, Searle RF (1998) Phenotypic and functional studies of leukocytes in human endometrium and endometriosis. Hum Reprod Update 4:702–709 10027623 10.1093/humupd/4.5.702

[75] Yang JH, Chen MJ, Chen HF, Lee TH, Ho HN, Yang YS (2004) Decreased expression of killer cell inhibitory receptors on natural killer cells in eutopic endometrium in women with adenomyosis. Hum Reprod 19:1974–1978 15217996 10.1093/humrep/deh372

[76] Zhihong N, Yun F, Pinggui Z, Sulian Z, Zhang A (2016) Cytokine profiling in the Eutopic Endometrium of Adenomyosis during the Implantation Window after ovarian stimulation. Reprod Sci 23:124–133 26239388 10.1177/1933719115597761

[77] Hierweger AM, Engler JB, Friese MA, Reichardt HM, Lydon J, DeMayo F, Mittrücker HW, Arck PC (2019) Progesterone modulates the T-cell response via glucocorticoid receptor-dependent pathways. Am J Reprod Immunol 81:e13084 30604567 10.1111/aji.13084 PMC7457140

[78] Southcombe JH, Mounce G, McGee K, Elghajiji A, Brosens J, Quenby S, Child T, Granne I (2017) An altered endometrial CD8 tissue resident memory T cell population in recurrent miscarriage. Sci Rep 7:41335 28112260 10.1038/srep41335 PMC5256279

[79] Feyaerts D, Benner M, van Cranenbroek B, van der Heijden OWH, Joosten I, van der Molen RG (2017) Human uterine lymphocytes acquire a more experienced and tolerogenic phenotype during pregnancy. Sci Rep 7 10.1038/s41598-017-03191-0 PMC5460245 28588205

[80] Wira CR, Fahey JV, Rodriguez-Garcia M, Shen Z, Patel MV (2014) Regulation of mucosal immunity in the Female Reproductive Tract: the role of sex hormones in Immune Protection against sexually transmitted pathogens. Am J Reprod Immunol 72:236–258 24734774 10.1111/aji.12252 PMC4351777

[81] White HD, Crassi KM, Givan AL, Stern JE, Gonzalez JL, Memoli VA, Green WR, Wira CR (1997) CD3 + CD8 + CTL activity within the human female reproductive tract: influence of stage of the menstrual cycle and menopause. J Immunol 158:3017–3027 9058841

[82] Scaife PJ, Bulmer JN, Robson SC, Innes BA, Searle RF (2006) Effector activity of decidual CD8 + T lymphocytes in early human pregnancy. Biol Reprod 75:562–567 16822900 10.1095/biolreprod.106.052654

[83] Ahmadi M, Abdolmohammadi-Vahid S, Ghaebi M, Aghebati-Maleki L, Afkham A, Danaii S, Abdollahi-Fard S, Heidari L, Jadidi-Niaragh F, Younesi V, Nouri M, Yousefi M (2017) Effect of intravenous immunoglobulin on Th1 and Th2 lymphocytes and improvement of pregnancy outcome in recurrent pregnancy loss (RPL). Biomed Pharmacother 92:1095–1102 28622710 10.1016/j.biopha.2017.06.001

[84] Saito S, Nakashima A, Shima T, Ito M (2010) Th1/Th2/Th17 and regulatory T-cell paradigm in pregnancy. Am J Reprod Immunol 63:601–610 20455873 10.1111/j.1600-0897.2010.00852.x

[85] Yu S, Diao L, Lian R, Chen C, Huang C, Li X, Li Y, Zeng Y (2023) Comparing the peri-implantation endometrial T-bet/GATA3 ratio between control fertile women and patients with recurrent miscarriage: establishment and application of a reference range. Hum Reprod 38:1680–1689 37353913 10.1093/humrep/dead132

[86] Tsuda S, Zhang X, Hamana H, Shima T, Ushijima A, Tsuda K, Muraguchi A, Kishi H, Saito S (2018) Clonally Expanded Decidual Effector Regulatory T Cells Increase in late Gestation of normal pregnancy, but not in Preeclampsia, in humans. Front Immunol 9 10.3389/fimmu.2018.01934 PMC6118230 30197648

[87] Nakashima A, Ito M, Yoneda S, Shiozaki A, Hidaka T, Saito S (2010) SHORT COMMUNICATION: circulating and decidual Th17 cell levels in healthy pregnancy. Am J Reprod Immunol 63:104–109 20015328 10.1111/j.1600-0897.2009.00771.x

[88] Manchorova D, Papadopoulou M, Alexandrova M, Dimitrova V, Djerov L, Zapryanova S, Dimitrova P, Vangelov I, Vermijlen D, Dimova T (2022) Human decidual gamma/delta T cells possess unique effector and TCR repertoire profiles during pregnancy. Cell Immunol 382 10.1016/j.cellimm.2022.104634 36308817

[89] Cai D, Tang Y, Yao X (2019) Changes of γδT cell subtypes during pregnancy and their influences in spontaneous abortion. J Reprod Immunol 131:57–62 30710888 10.1016/j.jri.2019.01.003

[90] Bulmer JN, Jones RK, Searle RF (1998) Intraepithelial leukocytes in endometriosis and adenomyosis: comparison of eutopic and ectopic endometrium with normal endometrium. Hum Reprod 13:2910–2915 9804254 10.1093/humrep/13.10.2910

[91] Kisovar A, Becker CM, Granne I, Southcombe JH (2023) The role of CD8 + T cells in endometriosis: a systematic review. Front Immunol 14:1225639 10.3389/fimmu.2023.1225639 PMC10366819 37497226

[92] Wu X-G, Chen J-J, Zhou H-L, Wu Y, Lin F, Shi J, Wu H-Z, Xiao H-Q, Wang W (2021) Identification and validation of the signatures of infiltrating immune cells in the eutopic endometrium endometria of women with endometriosis. Front Immunol 12:671201 10.3389/fimmu.2021.671201 PMC8446207 34539624

[93] Ahn SH, Edwards AK, Singh SS, Young SL, Lessey BA, Tayade C (2015) IL-17A contributes to the pathogenesis of endometriosis by triggering Proinflammatory cytokines and angiogenic growth factors. J Immunol 195:2591–2600 26259585 10.4049/jimmunol.1501138 PMC4561197

[94] Huang X, Wu L, Pei T, Liu D, Liu C, Luo B, Xiao L, Li Y, Wang R, Ouyang Y, Zhu H, Huang W (2023) Single-cell transcriptome analysis reveals endometrial immune microenvironment in minimal/mild endometriosis. Clin Exp Immunol 212:285–295 36869723 10.1093/cei/uxad029 PMC10243848

[95] Berbic M, Hey-Cunningham AJ, Ng C, Tokushige N, Ganewatta S, Markham R, Russell P, Fraser IS (2010) The role of Foxp3 + regulatory T-cells in endometriosis: a potential controlling mechanism for a complex, chronic immunological condition. Hum Reprod 25:900–907 20150173 10.1093/humrep/deq020

[96] Hey-Cunningham AJ, Riaz A, Fromm PD, Kupresanin F, Markham R, McGuire HM (2021) Circulating and Endometrial Regulatory T Cell and related populations in endometriosis and infertility: endometriosis is Associated with Blunting of Endometrial Cyclical effects and reduced proportions in moderate-severe disease. Reproductive Sci 29:229–242 10.1007/s43032-021-00658-4 34160778

[97] Scheerer C, Bauer P, Chiantera V, Sehouli J, Kaufmann A, Mechsner S (2016) Characterization of endometriosis-associated immune cell infiltrates (EMaICI). Arch Gynecol Obstet 294:657–664 27358184 10.1007/s00404-016-4142-6

[98] Ota H, Igarashi S, Tanaka T (1996) Expression of gamma delta T cells and adhesion molecules in endometriotic tissue in patients with endometriosis and adenomyosis. Am J Reprod Immunol 35:477–482 8738719 10.1111/j.1600-0897.1996.tb00128.x

[99] Gui T, Chen C, Zhang Z, Tang W, Qian R, Ma X, Cao P, Wan G (2014) The disturbance of TH17-Treg cell balance in adenomyosis. Fertil Steril 101:506–514 24331831 10.1016/j.fertnstert.2013.10.050

[100] Morita K, Tsuda S, Kobayashi E, Hamana H, Tsuda K, Shima T, Nakashima A, Ushijima A, Kishi H, Saito S (2020) Analysis of TCR repertoire and PD-1 expression in Decidual and peripheral CD8 + T cells reveals distinct Immune mechanisms in Miscarriage and Preeclampsia. Front Immunol 11 10.3389/fimmu.2020.01082 PMC7283903 32582176

[101] Yu S, Huang C, Lian R, Diao L, Zhang X, Cai S, Wei H, Chen C, Li Y, Zeng Y (2023) Establishment of reference intervals of endometrial immune cells during the mid-luteal phase. J Reprod Immunol 156:103822 36758471 10.1016/j.jri.2023.103822

[102] Tang C, Hu W (2023) The role of Th17 and Treg cells in normal pregnancy and unexplained recurrent spontaneous abortion (URSA): new insights into immune mechanisms. Placenta 142:18–26 37603948 10.1016/j.placenta.2023.08.065

[103] Sasaki Y (2004) Decidual and peripheral blood CD4 + CD25 + regulatory T cells in early pregnancy subjects and spontaneous abortion cases. Mol Hum Reprod 10:347–353 14997000 10.1093/molehr/gah044

[104] Sauerbrun-Cutler MT, Huber WJ, Krueger PM, Sung CJ, Has P, Sharma S (2021) Do endometrial natural killer and regulatory T cells differ in infertile and clinical pregnancy patients? An analysis in patients undergoing frozen embryo transfer cycles. Am J Reprod Immunol 85 10.1111/aji.13393 33501767

[105] Schulke L, Berbic M, Manconi F, Tokushige N, Markham R, Fraser IS (2009) Dendritic cell populations in the eutopic and ectopic endometrium of women with endometriosis. Hum Reprod 24:1695–1703 19321495 10.1093/humrep/dep071

[106] Mathur S, Peress MR, Williamson HO, Youmans CD, Maney SA, Garvin AJ, Rust PF, Fudenberg HH (1982) Autoimmunity to endometrium and ovary in endometriosis. Clin Exp Immunol 50:259–266 6759000 PMC1536699

[107] Arici A (2002) Local cytokines in endometrial tissue: the role of interleukin-8 in the pathogenesis of endometriosis. *Ann N Y Acad Sci* 955: 101-9; discussion 18, 396–406 10.1111/j.1749-6632.2002.tb02770.x 11949939

[108] Morimune A, Kimura F, Nakamura A, Kitazawa J, Takashima A, Amano T, Kaku S, Moritani S, Kushima R, Murakami T (2021) The effects of chronic endometritis on the pregnancy outcomes. Am J Reprod Immunol 85:e13357 33020952 10.1111/aji.13357

[109] Critchley HOD, Takebayashi A, Kimura F, Kishi Y, Ishida M, Takahashi A, Yamanaka A, Takahashi K, Suginami H, Murakami T (2014) The association between endometriosis and chronic endometritis. PLoS ONE 9:e88354 10.1371/journal.pone.0088354 PMC3928198 24558386

[110] Cicinelli E, Trojano G, Mastromauro M, Vimercati A, Marinaccio M, Mitola PC, Resta L, de Ziegler D (2017) Higher prevalence of chronic endometritis in women with endometriosis: a possible etiopathogenetic link. Fertil Steril 108: 289– 95.e1 10.1016/j.fertnstert.2017.05.016 28624114

[111] Khan KN, Fujishita A, Ogawa K, Koshiba A, Mori T, Itoh K, Nakashima M, Kitawaki J (2021) Occurrence of chronic endometritis in different types of human adenomyosis. Reproductive Med Biology 21 10.1002/rmb2.12421 PMC8967303 35386364

[112] Li J, Wei J, Chen S, Wang X, Chen J, Zeng D, Fan L (2024) Prevalence and risk factors for chronic endometritis in patients with adenomyosis and infertility: a retrospective cohort study. BMC Womens Health 24 10.1186/s12905-024-03245-2 PMC11251133 39014375

[113] Qiao X, Wu L, Liu D, Pei T, Huang W (2022) Existence of chronic endometritis and its influence on pregnancy outcomes in infertile women with minimal/mild endometriosis. Int J Gynecol Obstet 160:628–634 10.1002/ijgo.14326 35780459

[114] Lin S, Xie X, Chen Y, Wang Z, Zhang J, Liu C, Lin G, Wang Y, Guo Y (2024) How does chronic endometritis influence pregnancy outcomes in endometriosis associated infertility? A retrospective cohort study. Reproductive Health 21 10.1186/s12978-024-01897-9 PMC11566656 39543649

[115] Li Y, Yu S, Huang C, Lian R, Chen C, Liu S, Li L, Diao L, Markert UR, Zeng Y (2020) Evaluation of peripheral and uterine immune status of chronic endometritis in patients with recurrent reproductive failure. Fertil Steril 113: 187– 96.e1 10.1016/j.fertnstert.2019.09.001 31718829

[116] Matteo M, Cicinelli E, Greco P, Massenzio F, Baldini D, Falagario T, Rosenberg P, Castellana L, Specchia G, Liso A (2009) ORIGINAL ARTICLE: abnormal pattern of lymphocyte subpopulations in the Endometrium of Infertile Women with Chronic Endometritis. Am J Reprod Immunol 61:322–329 19341383 10.1111/j.1600-0897.2009.00698.x

[117] Chen X, Liu Y, Zhao Y, Cheung WC, Zhang T, Qi R, Chung JPW, Wang CC, Li TC (2020) Association between chronic endometritis and uterine natural killer cell density in women with recurrent miscarriage: clinical implications. J Obstet Gynecol Res 46:858–863 32189458 10.1111/jog.14250

[118] Kitazawa J, Kimura F, Nakamura A, Morimune A, Hanada T, Amano T, Tsuji S, Kasahara K, Satooka H, Hirata T, Kushima R, Murakami T (2020) Alteration in endometrial helper T-cell subgroups in chronic endometritis. Am J Reprod Immunol 85 10.1111/aji.13372 33155317

[119] Moreno I, Codoñer FM, Vilella F, Valbuena D, Martinez-Blanch JF, Jimenez-Almazán J, Alonso R, Alamá P, Remohí J, Pellicer A, Ramon D, Simon C (2016) Evidence that the endometrial microbiota has an effect on implantation success or failure. Am J Obstet Gynecol 215:684–703 27717732 10.1016/j.ajog.2016.09.075

[120] Akiyama K, Nishioka K, Khan KN, Tanaka Y, Mori T, Nakaya T, Kitawaki J (2019) Molecular detection of microbial colonization in cervical mucus of women with and without endometriosis. Am J Reprod Immunol 82 10.1111/aji.13147 31087436

[121] Wessels JM, Domínguez MA, Leyland NA, Agarwal SK, Foster WG (2021) Endometrial microbiota is more diverse in people with endometriosis than symptomatic controls. Sci Rep 11 10.1038/s41598-021-98380-3 PMC8460742 34556738

[122] Lin Q, Duan H, Wang S, Guo Z, Wang S, Chang Y, Chen C, Shen M, Shou H, Zhou C (2023) Endometrial microbiota in women with and without adenomyosis: a pilot study. Front Microbiol 14:1075900 10.3389/fmicb.2023.1075900 PMC9895119 36744089

[123] Yang W, Cong Y (2021) Gut microbiota-derived metabolites in the regulation of host immune responses and immune-related inflammatory diseases. Cell Mol Immunol 18:866–877 33707689 10.1038/s41423-021-00661-4 PMC8115644

[124] Xiong W, Zhang L, Yu L, Xie W, Man Y, Xiong Y, Liu H, Liu Y (2015) Estradiol promotes cells invasion by activating β-catenin signaling pathway in endometriosis. Reproduction 150:507–516 26432349 10.1530/REP-15-0371 PMC4633770

[125] Kitawaki J (2006) Adenomyosis: the pathophysiology of an oestrogen-dependent disease. Best Pract Res Clin Obstet Gynecol 20:493–502 10.1016/j.bpobgyn.2006.01.010 16564227

[126] Attar E, Tokunaga H, Imir G, Yilmaz MB, Redwine D, Putman M, Gurates B, Attar R, Yaegashi N, Hales DB, Bulun SE (2009) Prostaglandin E2 Via Steroidogenic Factor-1 Coordinately regulates transcription of steroidogenic genes necessary for Estrogen Synthesis in Endometriosis. J Clin Endocrinol Metabolism 94:623–631 10.1210/jc.2008-1180 PMC2646521 19001523

[127] Stewart J, Bulmer J, Murdoch A (1998) Endometrial leucocytes: expression of steroid hormone receptors. J Clin Pathol 51:121–126 9602685 10.1136/jcp.51.2.121 PMC500506

[128] Kang S, Wu Q, Yang B, Wu C (2022) Estrogen enhanced the expression of IL-17 by tissue‐resident memory γδT cells from uterus via interferon regulatory factor 4. FASEB J 36:e22166 10.1096/fj.202101443RR 35064703

[129] Kovats S (2015) Estrogen receptors regulate innate immune cells and signaling pathways. Cell Immunol 294:63–69 25682174 10.1016/j.cellimm.2015.01.018 PMC4380804

[130] Khan D, Ansar Ahmed S (2016) The immune system is a natural target for estrogen action: opposing effects of estrogen in two prototypical autoimmune diseases. Front Immunol 6:635 10.3389/fimmu.2015.00635 PMC4701921 26779182

[131] Polanczyk MJ, Carson BD, Subramanian S, Afentoulis M, Vandenbark AA, Ziegler SF, Offner H (2004) Cutting Edge: Estrogen drives expansion of the CD4 + CD25 + Regulatory T Cell Compartment. J Immunol 173:2227–2230 15294932 10.4049/jimmunol.173.4.2227

[132] Maret A, Coudert JD, Garidou L, Foucras G, Gourdy P, Krust A, Dupont S, Chambon P, Druet P, Bayard F, Guéry JC (2003) Estradiol enhances primary antigen-specific CD4 T cell responses and Th1 development in vivo. Essential role of estrogen receptor α expression in hematopoietic cells. Eur J Immunol 33:512–521 12645950 10.1002/immu.200310027

[133] Peixoto P, Cartron PF, Serandour AA, Hervouet E (2020) From 1957 to nowadays: a brief history of Epigenetics. Int J Mol Sci 21 10.3390/ijms21207571 PMC7588895 33066397

[134] Ray D, Yung R (2018) Immune senescence, epigenetics and autoimmunity. Clin Immunol 196:59–63 29654845 10.1016/j.clim.2018.04.002 PMC6548177

[135] Grimstad FW, Decherney A (2017) A review of the epigenetic contributions to endometriosis. Clin Obstet Gynecol 60:467–476 28742579 10.1097/GRF.0000000000000298

[136] Vannuccini S, Tosti C, Carmona F, Huang SJ, Chapron C, Guo SW, Petraglia F (2017) Pathogenesis of adenomyosis: an update on molecular mechanisms. Reprod Biomed Online 35:592–601 28693952 10.1016/j.rbmo.2017.06.016

[137] Szukiewicz D (2022) Epigenetic regulation and T-cell responses in endometriosis - something other than autoimmunity. Front Immunol 13:943839 35935991 10.3389/fimmu.2022.943839 PMC9355085

[138] Rocha-Junior CV, Da Broi MG, Miranda-Furtado CL, Navarro PA, Ferriani RA, Meola J (2019) Progesterone receptor B (PGR-B) is partially methylated in Eutopic Endometrium from Infertile Women with endometriosis. Reprod Sci 26:1568–1574 30782101 10.1177/1933719119828078

[139] Jichan N, Xishi L, Guo SW (2010) Promoter hypermethylation of progesterone receptor isoform B (PR-B) in adenomyosis and its rectification by a histone deacetylase inhibitor and a demethylation agent. Reprod Sci 17:995–1005 20697142 10.1177/1933719110377118

[140] Barnhart K, Dunsmoor-Su R, Coutifaris C (2002) Effect of endometriosis on in vitro fertilization. Fertil Steril 77:1148–1155 12057720 10.1016/s0015-0282(02)03112-6

[141] Horton J, Sterrenburg M, Lane S, Maheshwari A, Li TC, Cheong Y (2019) Reproductive, obstetric, and perinatal outcomes of women with adenomyosis and endometriosis: a systematic review and meta-analysis. Hum Reprod Update 25:592–632 31318420 10.1093/humupd/dmz012

[142] Wang JQ, Zhang JM, Qian B (2021) Adverse pregnancy outcomes for women with endometriosis: a systematic review and meta-analysis. Ginekol Pol 95:668–676 10.5603/GP.a2021.0081 34541648

[143] Huang Y, Zhao X, Chen Y, Wang J, Zheng W, Cao L (2020) Miscarriage on Endometriosis and Adenomyosis in Women by Assisted Reproductive Technology or with Spontaneous Conception: A Systematic Review and Meta-Analysis. *Biomed Res Int* 2020: 4381346 10.1155/2020/4381346 PMC7787757 33490243

[144] Bruun MR, Arendt LH, Forman A, Ramlau-Hansen CH (2018) Endometriosis and adenomyosis are associated with increased risk of preterm delivery and a small-for-gestational-age child: a systematic review and meta-analysis. Acta Obstet Gynecol Scand 97:1073–1090 29753309 10.1111/aogs.13364

[145] Lalani S, Choudhry AJ, Firth B, Bacal V, Walker M, Wen SW, Singh S, Amath A, Hodge M, Chen I (2018) Endometriosis and adverse maternal, fetal and neonatal outcomes, a systematic review and meta-analysis. Hum Reprod 33:1854–1865 30239732 10.1093/humrep/dey269 PMC6145420

[146] Qu H, Du Y, Yu Y, Wang M, Han T, Yan L (2022) The effect of endometriosis on IVF/ICSI and perinatal outcome: a systematic review and meta-analysis. J Gynecol Obstet Hum Reprod 51:102446 35905901 10.1016/j.jogoh.2022.102446

[147] Nagase Y, Matsuzaki S, Ueda Y, Kakuda M, Kakuda S, Sakaguchi H, Maeda M, Hisa T, Kamiura S (2022) Association between Endometriosis and Delivery Outcomes: A Systematic Review and Meta-Analysis. *Biomedicines* 10 10.3390/biomedicines10020478 PMC8962356 35203685

[148] Cozzolino M, Tartaglia S, Pellegrini L, Troiano G, Rizzo G, Petraglia F (2022) The effect of uterine adenomyosis on IVF outcomes: a systematic review and Meta-analysis. Reprod Sci 29:3177–3193 34981458 10.1007/s43032-021-00818-6

